# Prediction of acute respiratory infections using machine learning techniques in Amhara Region, Ethiopia

**DOI:** 10.1038/s41598-024-76847-3

**Published:** 2024-11-14

**Authors:** Abdulaziz Kebede Kassaw, Gashaw Bekele, Ahmed Kebede Kassaw, Ali Yimer

**Affiliations:** 1 Department of Health Informatics, School of Public Health, College of Medicine and Health Sciences, Wollo University, Dessie, Ethiopia; 2 Department of Software Engineering, College of Informatics, Kombolcha Institute of Technology, Wollo University, Kombolcha, Ethiopia; 3 Department of Information System, College of Informatics, Kombolcha Institute of Technology, Wollo University, Kombolcha, Ethiopia; 4 Department of Public Health, College of Health Sciences, Woldia University, Woldia, Ethiopia

**Keywords:** Diseases, Health care

## Abstract

Many studies have shown that infectious diseases are responsible for the majority of deaths in children under five. Among these children, Acute Respiratory Infections is the most prevalent illness and cause of death worldwide. Acute respiratory infections continue to be the leading cause of death in developing countries, including Ethiopia. In order to predict the main factors contributing to acute respiratory infections in the Amhara regional state of Ethiopia, a machine learning technique was employed. This study utilized data from the 2016 Ethiopian Demographic and Health Survey. Seven machine learning models, including logistic regression, random forests, decision trees, Gradient Boosting, support vector machines, Naïve Bayes, and K-nearest neighbors, were employed to forecast the factors influencing acute respiratory infections. The accuracy of each model was assessed using receiver operating characteristic curves and various metrics. Among the seven models used, the Random Forest algorithm demonstrated the highest accuracy in predicting acute respiratory infections, with an accuracy rate of 90.35% and Area under the Curve of 94.80%. This was followed by the Decision Tree model with an accuracy rate of 88.69%, K-nearest neighbors with 86.35%, and Gradient Boosting with 82.69%. The Random Forest algorithm also exhibited positive and negative predictive values of 92.22% and 88.83%, respectively. Several factors were identified as significantly associated with ARI among children under five in the Amhara regional state, Ethiopia. These factors, included families with a poorer wealth status (log odds of 0.18) compared to their counterparts, families with four to six children (log odds of 0.1) compared to families with fewer than three living children, children without a history of diarrhea (log odds of -0.08), mothers who had occupation(log odds of 0.06) compared mothers who didn’t have occupation, children under six months of age (log odds of -0.05) compared to children older than six months, mothers with no education (log odds of 0.04) compared to mothers with primary education or higher, rural residents (log odds of 0.03) compared to non-rural residents, families using wood as a cooking material (log odds of 0.03) compared to those using electricity. Through Shapley Additive exPlanations value analysis on the Random Forest algorithm, we have identified significant risk factors for acute respiratory infections among children in the Amhara regional state of Ethiopia. The study found that the family’s wealth index, the number of children in the household, the mother’s occupation, the mother’s educational level, the type of residence, and the fuel type used for cooking were all associated with acute respiratory infections. Additionally, the research emphasized the importance of children being free from diarrhea and living in households with fewer children as essential factors for improving children’s health outcomes in the Amhara regional state, Ethiopia.

## Introduction

Preventable infectious diseases continue to require special attention even though child mortality rates have declined globally over the past few decades. Infectious diseases account for 68% of deaths in children under five, making them the primary cause of death in this age group^1^.

The major causes of death for children under the age of five, according to the Global Health Observatory (GHO) 2016 report, were issues associated to preterm birth, Pneumonia, complications related to childbirth, congenital malformations, and diarrhea. Pneumonia makes it difficult to breathe normally^2^. According to the site of infection, there are two types of Pneumonia (acute respiratory infections): acute upper respiratory infections and acute lower respiratory infections^3^.

Acute respiratory infections (ARIs) remain the second most common cause of illness and death in children under five worldwide. Worldwide, hospitals admit over 12 million children with severe acute respiratory illnesses each year^5^. The majority of ARI deaths among children under five were concentrated in Sub-Saharan Africa and Southeast Asia. ARIs continues to be the largest burden in developing countries, including Ethiopia^4^. Ethiopia is among the top 15 countries with the highest burden of acute respiratory infections (ARIs), affecting about 3.4 million children annually^6^. It kills over 40,000 children under the age of five annually and accounts for 18% of all deaths^7^.

There are notable regional and national differences in the prevalence of acute respiratory illness (ARI) in children under five. These differences can be attributed to the mother, the child, environmental factors, and co-occurring diseases like diarrhea, measles, and malaria^8^.

Conventional regression models have been used in the past to conduct extensive research on the socioeconomic and demographic factors associated with acute respiratory infections in Ethiopia^9–15^ [9–15]. Numerous studies looked at the factors that determine the prevalence of acute respiratory infections in children under five years old in different parts of Ethiopia using cross-sectional statistical methodologies. The logistic regression methods used were both bivariate and multivariate. Finding novel and unexpected patterns in data, as well as hidden cause and effect linkages, can be challenging for cross-sectional statistical approaches. Most of the previous research was focused on a limited number of risk variables, used small data sets of fewer than 600 cases or records, and was limited to a single city or town for its local clinical data.

This study predicts the important factors associated with acute respiratory infection in the Amhara regional state of Ethiopia using non-classical regression models and nationally representative data. More specifically, the study sample’s acute respiratory infection risk factors were predicted using machine-learning techniques. Determining risk indicators, creating a prediction model, and extracting relevant rules for users and decision-makers were the steps taken in this work to fill in the gaps left by the traditional approaches.

## Methods

### Study design and setting

A cross-sectional study was conducted on children under the age of five in the Amhara region of Ethiopia. The researchers used data from the 2016 Ethiopian Demographic and Health Survey for their analysis.

### Sample size estimation and sampling techniques

This was a cross-sectional study conducted between January 18 and June 27, 2016. The data came from the 2016 Ethiopian Demographic and Health Survey (EDHS), which was the fourth time Ethiopia participated in this global survey program. The data was collected using a standardized, previously validated questionnaire, with interviewers recording responses on tablet computers. Bluetooth technology was used to enable remote electronic file transmission. The EDHS selected a representative sample of over 18,000 households across 624 clusters in Ethiopia’s nine regions and two administrative cities. A two-stage cluster sampling design was used, first selecting census enumeration areas and then households within those areas. All women aged 15–49 with at least one child under 5 were eligible to participate. The final sample size for this specific study focused on the Amhara region was 4,638 children under five, after removing cases with missing data.

### Criteria for exclusion and inclusion

The data used in this study was from the under-5 children population in the 2016 Ethiopian Demographic and Health Survey (EDHS) dataset. Participants with incomplete information or missing values were excluded from the analysis for this research.

### Study variables and measurements

The outcome variable in this study was the presence or absence of acute respiratory illness (ARI) in children under 5 years old. ARI was coded as one to indicate presence and zero to indicate absence. A child was considered to have ARI if the mother reported the child had cough and rapid breathing in the last two weeks. The diagnosis of ARI was based on the mother’s report of the child’s symptoms^11^. The variables included in the analysis were selected based on their availability in the EDHS dataset and their established theoretical relationship to the outcome of interest, as documented in the existing literature. The variables were used in the analysis in their original coded form as they appeared in the EDHS data^10,]^^23–26^. In addition to the variables available in the original EDHS dataset, the researchers also derived several new variables for the analysis. These included Age of child (less than 6months, place of residence (urban, rural), maternal educational level (no education, primary, secondary and above), Cooking fuel (electricity, charcoal, wood), wealth quintile (poorest, poorer, middle, richer, richest), maternal occupation (not working, working), child sex (male, female), breast-feeding (never breast-feed, ever breastfeed not currently), Vitamin A supplementation (yes, no), recent diarrhea (no, yes) and number of living children (1–3 child, 4–6 child, above 6). The drinking water sources variable was recoded into two categories: “improved” or “unimproved”. This recoding was done based on standard definitions and classifications of drinking water sources^27^. Children’s nutritional status was assessed by calculating z-scores for “height-for-age (stunting)” and “weight-for-height (wasting)” using WHO-recommended child physical growth indicators^13,28^. Children were classified into different nutritional status categories based on their z-scores for height-for-age and weight-for-height, relative to the WHO reference population median. It is classified as follows; Normal growth: z-scores between − 2 and + 2; Stunted: height-for-age z-score (HAZ) < -2 and Wasted: weight-for-height z-score (WHZ) < -2.

These standard z-scores used to assess child growth are based on the WHO Child Growth Standards, which provide reference ranges for various anthropometric measurements^13,28^. The media exposure variable was created by access to either radio, TV or newspapers. If the child’s household had access to at least one of these media sources, the child was coded as “yes” for media exposure. If the household had no access to any of these media sources, the child was coded as “no” for media exposure.

### Data quality control

The data for this study was obtained from the secondary 2016 Ethiopian Demographic and Health Survey (EDHS) dataset. The data extraction process followed the required procedures very closely. Extensive data cleaning was carried out carefully to ensure data quality. The quality of the 2016 EDHS data was primarily determined by the quality of the fieldwork, which was enhanced through appropriate steps taken during data processing. Critical data entry and editing for inconsistencies were performed to remove any missing data. Each relevant and essential data pre-processing step was diligently carried out to assure the overall quality and integrity of the data used in the analysis.

### Data preprocessing

**Fig. 1:**
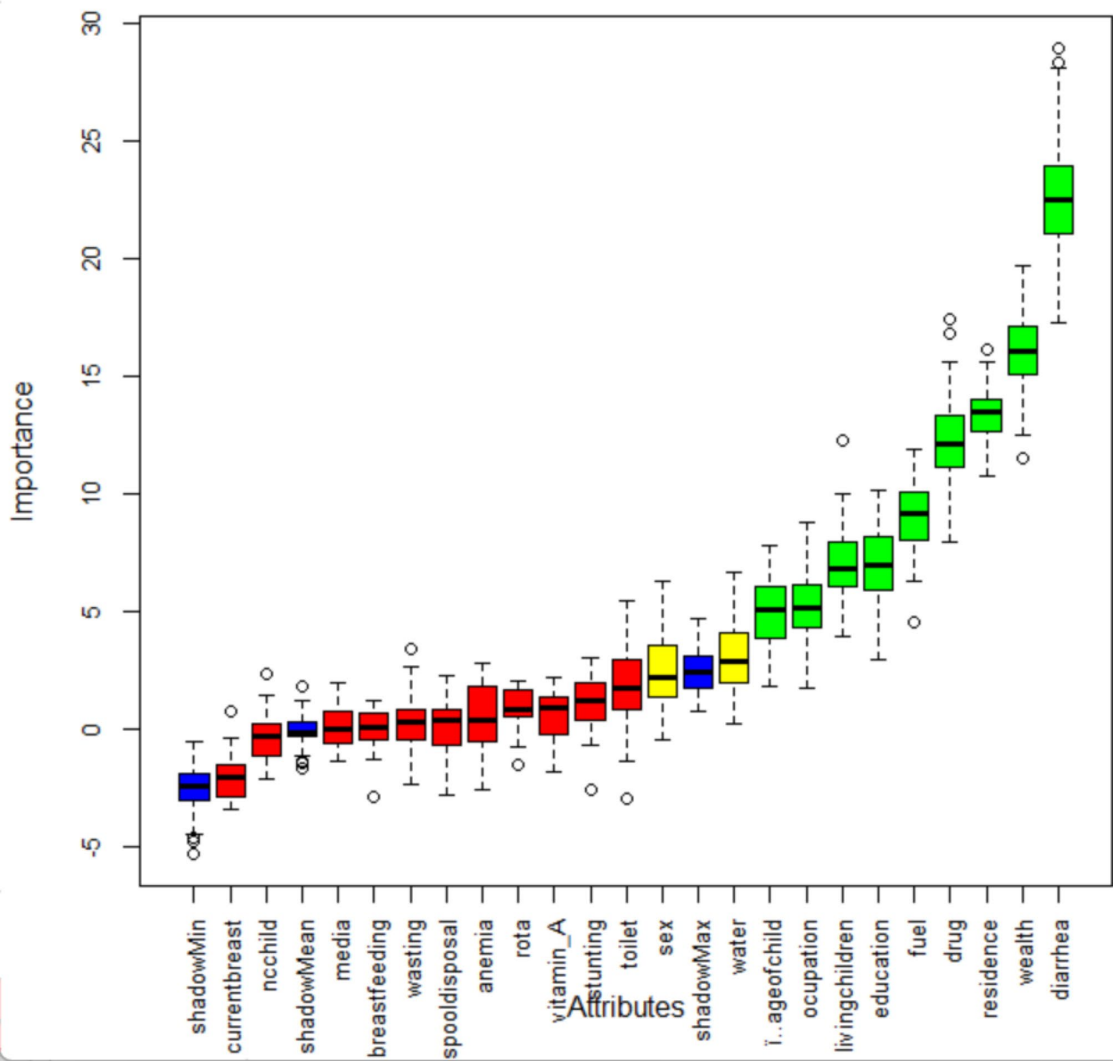
Feature selection using Boruta algorithm.

The dataset used in this study contained 23 features and 4,638 instances. Various data preprocessing techniques were applied to create a predictive model for Acute Respiratory Infection (ARI) among children under five in Ethiopia. These techniques included: First data cleaning in which Missing values in categorical data were imputed using mode imputation. The second technique was data transformation in which redundant data was manually removed. Features with multiple categorical values (e.g., drinking water source, BMI, wealth index, parents’ occupation, fuel type) were transformed into discrete values through binning discretization, which was necessary for the subsequent data mining tasks. The third was Class imbalance handling which used Appropriate techniques to address any imbalance in the target class (ARI presence/absence).The fourth was Feature selection in which the Boruta algorithm was employed to identify the most important features for the analysis (Fig. 1).

After the comprehensive data preprocessing steps, the final dataset contained 4,638 instances with 23 attributes. This prepared dataset was then used for the analysis and construction of the predictive model for Acute Respiratory Infection (ARI). To assess the model’s performance, the dataset was split into training and testing sets using an 80/20 ratio. However, the training dataset had an imbalanced class distribution, which could introduce bias in the results. To address this issue, the Synthetic Minority Over-sampling Technique (SMOTE) was used. This technique generates additional instances of the minority class, balancing the class distribution in the training dataset. This ensures that all classes are adequately represented during the model training process. Applying this class imbalance handling technique helps prevent the loss of important data and reduces potential bias in the final predictive model. These steps were crucial in preparing the data for building an accurate and reliable predictive model for Acute Respiratory Infection in the target population.

To create synthetic samples and address the class imbalance in the dataset, the Synthetic Minority Over-sampling Technique (SMOTE) was applied. SMOTE uses a k-nearest neighbors (KNN) algorithm to generate the synthetic samples^16,17^. The synthetic samples generated by SMOTE are not exact duplicates of the original minority class instances. Rather, they are new, similar data points created by interpolating between the existing minority class feature vectors and their nearest neighbors. This approach ensures that the synthetic samples added to the dataset are still representative of the underlying minority class distribution, but introduces some variability to improve the model’s ability to generalize^16,17^. To address the imbalance in the dataset, the Synthetic Minority Over-sampling Technique (SMOTE) algorithm was used. SMOTE generates new synthetic instances of the minority class by interpolating between the feature vectors of the minority class samples and their nearest neighbors. Applying the SMOTE algorithm rebalanced the dataset in two ways as follows. The number of cases (minority class) was increased from 209 to 3360 and the number of controls (majority class) was decreased from 4429 to 3,528. The analysis used a balanced dataset of 6,888 instances after applying SMOTE. This balanced dataset helped prevent model bias and improved the accuracy and reliability of the analysis by avoiding issues of under fitting or overfitting.

### Predictive model development

Predictive modeling is the process of creating a model that can forecast future results by studying past and present data. The model is developed by analyzing historical data to identify patterns and relationships, which are then used to make predictions about future outcomes[18]. The aim of this study was to develop a predictive model for Acute Respiratory Infection (ARI) among children under 5 years old in the Amhara region of Ethiopia. To achieve this, the researchers employed and compared several machine learning algorithms, including Decision Tree, Random Forest, K-Nearest Neighbor, Support Vector Machine (SVM), Naive Bayes, Gradient Boosting and Logistic Regression.

In order to optimize the performance of each algorithm, the researchers used Grid Search to fine-tune the hyper parameters of the models.

The goal was to identify the most accurate and robust predictive model for forecasting ARI cases in this population of young children. By testing multiple machine learning techniques, the researchers could determine the best-performing approach for this specific predictive modeling task. Tuning the hyper parameters of the machine learning algorithms is a crucial step in this study. The selection of the right hyper parameters greatly impacts the model’s performance^19–21^. To assess the performance of each predictive model, the researchers used several evaluation metrics, including Accuracy, Sensitivity, Specificity, Positive Predictive Value, Negative Predictive Value and ROC AUC (Receiver Operating Characteristic Area under Curve).

The researchers utilized a diverse set of evaluation metrics to comprehensively assess the performance of the different machine learning models. This allowed them to make a thorough comparison of the strengths and weaknesses of each modeling approach. Evaluating the models across multiple relevant metrics is important to get a holistic understanding of their predictive capabilities, robustness, and reliability. The data preprocessing and analysis for this study were conducted using the R programming language and the caret package^22^.

To evaluate the performance of the predictive models, the researchers randomly selected 80% of the sample data and used this 80% of the data to train the machine learning models. The researchers took the remaining 20% of the sample data that was not used for training the models, and they used this 20% for 10-fold cross-validation. Cross-validation is a technique used to fine-tune the models’ hyper parameters and evaluate their performance in an unbiased way. After using 80% of the data to train the models, and the remaining 20% for 10-fold cross-validation to fine-tune the hyper parameters, the researchers then used the test data from that 20% random sample to assess the final performance of the models. By calculating this comprehensive set of accuracy measures, the researchers were able to gain a holistic understanding of the models’ performance in correctly detecting ARI cases versus non-ARI cases. This provided valuable insights into the overall predictive capabilities, robustness, and real-world applicability of the different machine learning models. The researchers also utilized the area under the curve (AUC) and receiver operating characteristic (ROC) curve metrics to further evaluate the models’ ability to differentiate between instances containing Acute Respiratory Infection (ARI) and those without ARI. The ROC curve allows for a visualization of the model’s performance across different thresholds, while the AUC condenses this information into a single, easily interpretable numerical value. Together, these two metrics offer a comprehensive evaluation of the model’s suitability for the binary classification task. By analyzing both the ROC curve and the AUC, the researchers can gain deeper insights into the model’s strengths, weaknesses, and overall discriminative capabilities in distinguishing between the two classes (e.g., ARI-positive and ARI-negative instances)^23^. Therefore, a higher AUC value means the model is better able to differentiate the positive class (e.g., ARI-positive cases) from the negative class (e.g., ARI-negative cases). This is a crucial metric for evaluating the overall discriminative power and effectiveness of the classification model^23^.

To assess the relevance and importance of each variable in the machine learning models, the researchers computed the Mean Decrease in Gini metric. This measure provides an indication of how much each variable contributes to the overall splitting decisions made by the model during the training process. The Mean Decrease in Gini is a commonly used feature importance metric in tree-based models, such as Random Forests. It quantifies the degree to which a variable helps to reduce the Gini impurity, which is a measure of how well a potential split can separate the target classes. The researchers then used this Mean Decrease in Gini information to identify the top categories of variables that were most influential in the best predictive ML model. This was likely done through the creation of a graphical visualization that automatically highlighted the variables with the highest feature importance according to the Gini metric. By determining the key variables driving the model’s performance, the researchers were able to gain valuable insights into the underlying factors and patterns that were most predictive of the target outcome (e.g., distinguishing between ARI and non-ARI cases). This analysis of variable importance provides critical information for interpreting the model’s behavior, understanding the problem domain, and potentially guiding future feature engineering and model refinement efforts.

### Model interpretation/explanation using Shapley additive exPlanations (SHAP)

Shapley Additive exPlanations (SHAP) is an analysis technique based on game theory principles. It is used to explain and interpret the predictions made by any machine learning model, both globally (for the entire model) and locally (for individual predictions)^24^. In machine learning, it is challenging to explain high-performing “black-box” models like tree-based models. The fundamental concept behind SHAP analysis is to compute the marginal contribution of each predictor variable towards the outcome variable prediction. In this study, SHAP analysis was applied for two purposes: First, it was applied for feature selection. SHAP provides a unified feature importance measure based on Shapley values, which quantify each feature’s contribution to the predictions across the entire population. This SHAP-based feature selection method has been shown to improve classification performance while maintaining model explain ability^25,26^. The second purpose of using SHAP analysis in this study was to interpret the effect of each predictor on the prediction of ARIs (Acute Respiratory Infections). This was done by plotting the aggregate Shapley value for each feature across all the samples. This allowed the researchers to explain whether a particular feature increases or decreases a child’s likelihood of developing ARIs. The Shapley values provide insights into how each predictor variable contributes to the model’s predictions of the outcome.

The researchers used a SHAP waterfall plot to explain the contributions of each feature towards the prediction of the positive class (i.e. had ARI). The x-axis represents the probability of classifying a sample as the “ARI” class. The y-axis displays the independent variables and their corresponding feature values for that specific sample. A horizontal bar represents each feature’s contribution. Positive contributions (red bars) indicate the feature increases the likelihood of the sample belonging to the positive class. Negative contributions (blue bars) suggest the feature decreases the likelihood of the sample belonging to the positive class. By analyzing the SHAP waterfall plot, the researchers could gain insights into the relative importance and directionality of the different features in determining the classification outcome for a specific sample. This provides a detailed, interpretable view of the model’s decision-making process.

## Results

### Descriptive results of socio-demographic characteristics

Out of the 4638 study subjects, 81.0% of them were rural dwellers. Among these rural respondents, Acute Respiratory Infection (ARI) affected 5%, while the remaining 95% were not affected by ARI infection. In terms of the mother’s work status, 59.1% of the children’s mothers did not have a job. Regarding the educational status of the respondents’ mothers, approximately 63.4% of the participants had no education. When it comes to the wealth category of the respondents’ families, around 37.4% fell under the poorest category. In terms of the sex of the children in the study, the majority, 50.3%, were categorized as male (Table 1).

**Table 1:** Socio-demographic characteristics of respondents in Amhara, Ethiopia from January 18 to June 27, 2016 (*N* = 4638).

				ARI			
				NO		YES	
Variable	Category	Frequency	Column %	frequency	Row %	Frequency	Row %
age of child	< 6month/(0)	602	13.0%	579	96.2%	23	3.8%
	6-11month/(1)	380	8.2%	358	94.2%	22	5.8%
	12-23month/(2)	842	18.2%	791	93.9%	51	6.1%
	24-35month/(3)	940	20.3%	899	95.6%	41	4.4%
	36-47month/(4)	870	18.8%	829	95.3%	41	4.7%
	48-59month/(5)	1004	21.6%	973	96.9%	31	3.1%
sex of child	Male/(0)	2335	50.3%	2231	95.5%	104	4.5%
	Female/(1)	2303	49.7%	2198	95.4%	105	4.6%
type of place of residence	urban/(1)	883	19.0%	860	97.4%	23	2.6%
	rural/(2)	3755	81.0%	3569	95.0%	186	5.0%
highest educational level	no/(0)	2941	63.4%	2800	95.2%	141	4.8%
	Primary/(1)	1118	24.1%	1065	95.3%	53	4.7%
	Secondary/(2)	362	7.8%	353	97.5%	9	2.5%
	Higher/(3)	217	4.7%	211	97.2%	6	2.8%
wealth index combined	Poorest/(1)	1736	37.4%	1679	96.7%	57	3.3%
	Poorer/(2)	750	16.2%	709	94.5%	41	5.5%
	Middle/(3)	616	13.3%	575	93.3%	41	6.7%
	richer/(4)	558	12.0%	518	92.8%	40	7.2%
	Richest/(5)	978	21.1%	948	96.9%	30	3.1%
living children	1–3/(0)	2408	51.9%	2309	95.9%	99	4.1%
	4–6/(1)	1569	33.8%	1480	94.3%	89	5.7%
	above 6/(2)	661	14.3%	640	96.8%	21	3.2%
Occupation	Not working/(0)	2741	59.1%	2639	96.3%	102	3.7%
	working/(1)	1897	40.9%	1790	94.4%	107	5.6%

### Environmental characteristics of respondents

In this survey, it was found that 4151 participants (89.5%) did not practice safe disposal of stools when not using a toilet. Among these, 4.6% were infected by Acute Respiratory Infection (ARI), while the remaining 95.4% were not affected by ARI. The majority of respondents, 4030 (86.9%), used unimproved restrooms. Additionally, 3613 participants (77.9%) cooked with wood as their primary fuel source. Regarding media access, a large portion of the participants, 2937 (63.3%) did not have access to media (Table 2).

**Table 2:** Environmental characteristics of the respondents in Amhara,  Ethiopia from January 18 to June 27, 2016 (*N* = 4638).

				ARI			
				NO		YES	
Variable	Category	Frequency	Column %	Frequency	Row %	frequency	Row %
fuel type	electricity/(0)	285	6.1%	279	97.9%	6	2.1%
	charcoal/(1)	437	9.4%	423	96.8%	14	3.2%
	Wood/(2)	3613	77.9%	3441	95.2%	172	4.8%
	others/(3)	303	6.5%	286	94.4%	17	5.6%
Toilet	Improved/(0)	608	13.1%	589	96.9%	19	3.1%
	not improved/(1)	4030	86.9%	3840	95.3%	190	4.7%
Source of drinking water	Not improved /(0)	2559	55.2%	2440	95.3%	119	4.7%
	improved/(1)	2079	44.8%	1989	95.7%	90	4.3%
Media exposure	No/(0)	2937	63.3%	2807	95.6%	130	4.4%
	Yes/(1)	1701	36.7%	1622	95.4%	79	4.6%
Stools disposal when not using toilet	not safe/(0)	4151	89.5%	3958	95.4%	193	4.6%
	safe/(1)	487	10.5%	471	96.7%	16	3.3%

### Nutritional and co-morbid characteristics among under-five children

Out of the total participants, 2635 (56.8%) children had no history of diarrhea. Among these children, 3.2% were co-infected by Acute Respiratory Infection (ARI).Regarding breastfeeding, 4448 (95.9%) children were breastfed. In terms of nutritional status, 1912 (41.2%) children were stunted, indicating impaired growth, and 431 (9.3%) children were wasted, indicating acute malnutrition. The majority of children, 4094 (88.3%), did not receive any medication for intestinal parasites in the previous six months. Additionally, 5326 (56.0%) children did not receive vitamin A during that time (Table 3).

**Table 3:** Nutritional and co-morbid characteristics of ARI among under-five children in Amhara, Ethiopia from January 18 to June 27, 2016 (*N* = 4638).

				ARI			
				NO		YES	
Variable	Category	Frequency	Column %	frequency	Row %	frequency	Row %
Stunting	Normal/(0)	2726	58.8%	2611	95.8%	115	4.2%
	severe/(1)	1912	41.2%	1818	95.1%	94	4.9%
intestinal parasites drug	no/(0)	4094	88.3%	3921	95.8%	173	4.2%
	yes/(1)	544	11.7%	508	93.4%	36	6.6%
duration of breast feeding	ever /(0)	4448	95.9%	4242	95.4%	206	4.6%
	never/(1)	190	4.1%	187	98.4%	3	1.6%
wasting,	Normal /(0)	4207	90.7%	4018	95.5%	189	4.5%
	wasting/(1)	431	9.3%	411	95.4%	20	4.6%
Vitamin A supplement	no/(0)	2635	56.8%	2526	95.9%	109	4.1%
	yes/(1)	2003	43.2%	1903	95.0%	100	5.0%
Rotavirus- Vaccine	not /(0)	304	6.6%	294	96.7%	10	3.3%
	vaccinated/(1)	4334	93.4%	4135	95.4%	199	4.6%
Had Anemia	no/(0)	2863	61.7%	2736	95.6%	127	4.4%
	yes/(1)	1775	38.3%	1693	95.4%	82	4.6%
Media exposure	No/(0)	2937	63.3%	2807	95.6%	130	4.4%
	Yes/(1)	1701	36.7%	1622	95.4%	79	4.6%
Had diarrhea	no/(0)	4175	90.0%	4042	96.8%	133	3.2%
	yes/(1)	463	10.0%	387	83.6%	76	16.4%

### Feature selection

The selection of features is a critical phase in predictive modeling^27,28^. This step becomes particularly important when dealing with datasets containing numerous variables for model construction. In this study, we employed the Boruta feature selection algorithm, which is commonly used when the goal is to understand the mechanisms related to the variable of interest. This algorithm helps identify the most relevant features that contribute to the predictive model’s performance and provides insights into the variables’ relationships and importance (Fig. 1).

Using the Boruta feature selection method, nine variables out of the original twenty-three variables were identified as important features for model construction. Variables such as Diarrhea, Wealth, Residence, and fuel type were found to be significant for building the model, represented by the color blue. Variables like water and sex of the child were classified as tentative, meaning they had a mixed effect on the model and were represented by the color yellow. The model as unnecessary for the prediction task rejected the remaining attributes, including stunting, wasting, and media exposure. The color red, indicating they did not contribute significantly to the model’s performance and were excluded from the feature set (Fig. 1), represented these attributes.

### Predicting under-five children acute respiratory infection status

Among the seven models evaluated, the Random Forest algorithm achieved the highest accuracy in predicting Acute Respiratory Infection (ARI) with an accuracy value of 90.35%. It was followed by the Decision Tree model with an accuracy of 88.69%, K-nearest neighbor with 86.35%, and Gradient Boosting with 82.69%. For the Random Forest algorithm, the positive predictive value (PPV) was found to be 92.22% and the negative predictive value (NPV) was 88.83%. The sensitivity (true positive rate) was 87.76% and the specificity (true negative rate) was 92.93%. The outcomes of all seven machine learning models, including Decision Tree, Random Forest, Naïve Bayes, Support Vector Machine (SVM), K-nearest neighbor (KNN), Logistic Regression, and Gradient Boosting, are presented in detail below (Table 4).

**Table 4:** Metrics of model accuracy for each model as assessed using test data.

	Machine learning algorithms						
	Decision Tree	Random Forest	Naïve Bayes	Logistic Regression	KNN	SVM	Gradient Boosting
	%	%	%	%	%	%	%
**Accuracy**	88.69	90.35	69.06	68.93	86.35	79.52	82.69
**Sensitivity**	88.07	87.76	68.43	72.36	79.91	68.43	77.49
**Specificity**	89.32	92.93	68.70	65.51	92.78	90.62	87.88
**PP Value**	88.74	92.22	68.33	66.71	91.36	87.45	85.93
**NP Value**	88.68	88.83	69.80	71.21	82.86	75.03	80.34
**AUC**	79.20	94.80	73.50	73.20	90.90	80.40	81.20

AUC: Area under the curve, KNN: K-nearest neighbor, SVM: Support vector machine.

PP: Positive predictive, NP: Negative predictive.

### ROC curve for the tested models

**Fig. 2:**
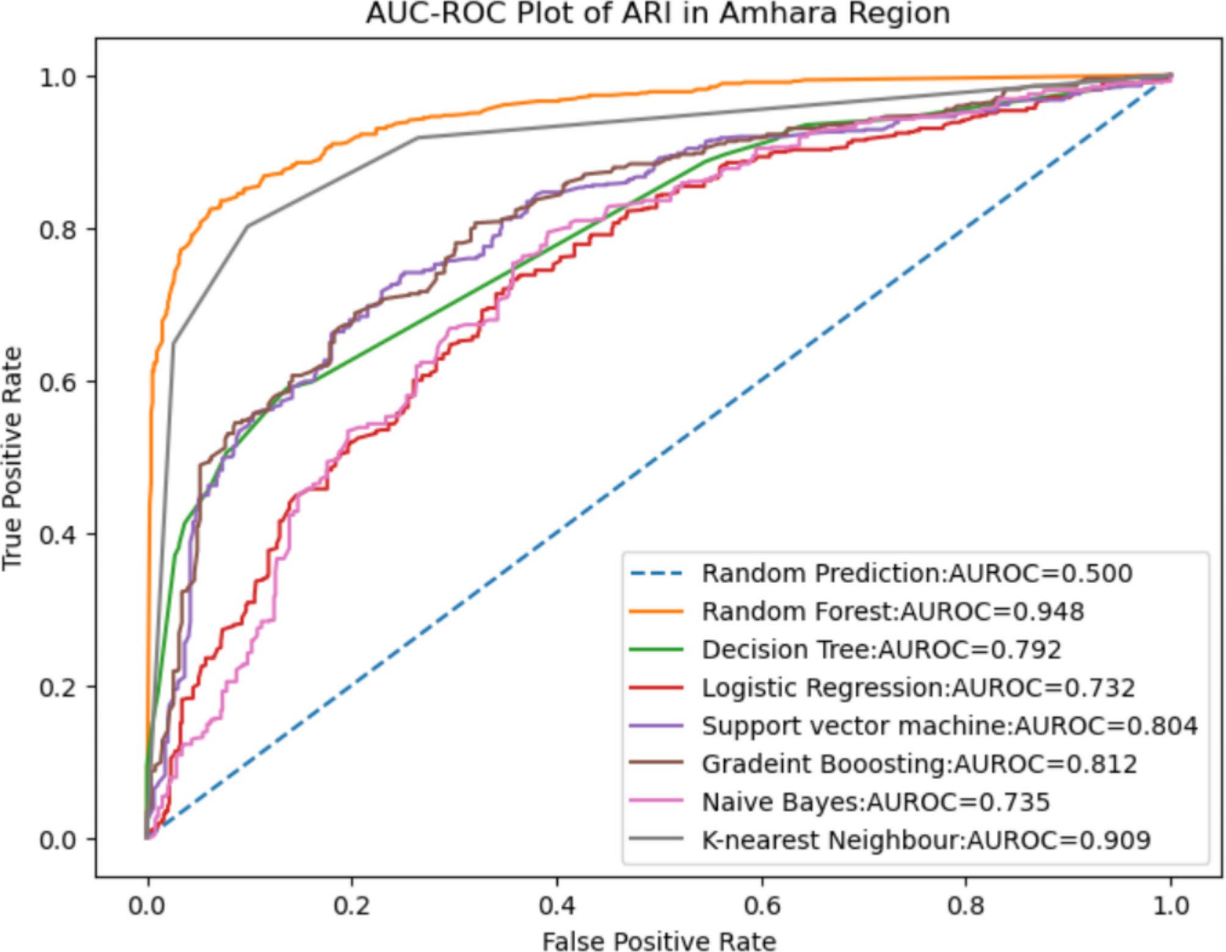
ROC curve for the seven models.

Figure 2 illustrates the receiver operating characteristics (ROC) curve, providing a visual representation of the model performance. Among the seven machine learning models used in this study, the Random Forest (RF) model exhibits the highest area under the curve (AUC) value on the ROC curve. The AUC is a robust performance measurement that demonstrates the model’s effectiveness in distinguishing between children with Acute Respiratory Infection (ARI) and those without. The RF model’s higher AUC value indicates its superior discriminatory power compared to other metrics such as accuracy, specificity, sensitivity, positive predictive value, and negative predictive value (Table 4).

**Fig. 3:**
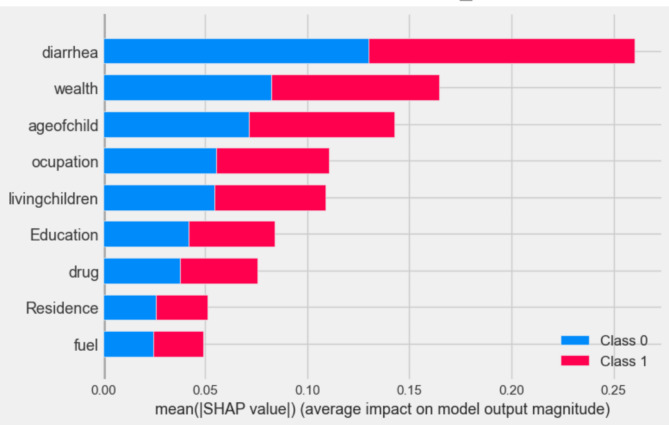
SHAP global importance plot of optimized Random Forest model Class 0 = no ARI; Class 1 = had ARI.

Based on the results, the next step is to determine the magnitude of predictor variables using the Random Forest model-based SHAP (Shapley Additive Explanations) values. The SHAP values provide insights into the importance and contribution of each predictor variable towards the model’s predictions (Fig. 3).

The SHAP global importance scores for the top nine factors, determined using the optimized random forest model, illustrate the contribution of global features towards predicting Acute Respiratory Infection (ARI). The predictors are sorted in descending order based on their impact on the prediction of the outcome variable. The results indicate that the most influential factors for predicting ARI are diarrhea, wealth, age of the child, numbers of living children in the same household, mother’s occupation, mother’s educational status, intake of intestinal drugs, type of residence, and type of fuel used for cooking in the household. These factors, ranked by their mean absolute SHAP values, play a significant role in determining the likelihood of ARI occurrence (Fig. 3).

#### Model interpretation and justification

**Fig. 4:**
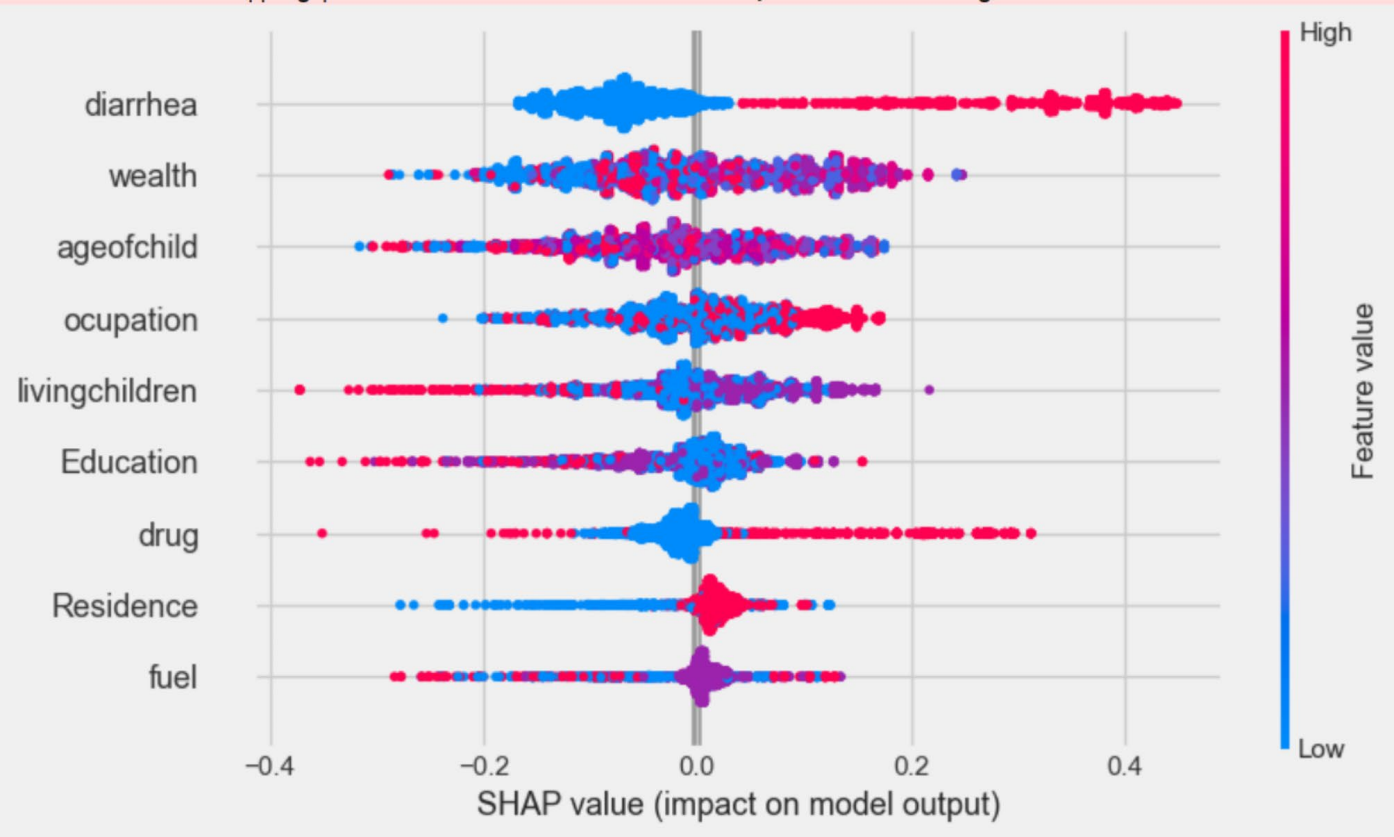
Beeswarm plot, ranked by mean absolute SHAP value generated by optimized Random Forest.

To give a comprehensive picture of how the variables affect the model’s predictions across the board, Beeswarm plots were employed. Figure 4 shows the distribution of each predictor’s effects on the output of the model (i.e. ARI prediction) by graphing each sample’s Shapley value for that specific predictor. The significance and correlation between each of the top nine features on the outcome variable are shown by the points on this beeswarm plot, which represent the Shapley values of the features linked to ARI infection. The higher and lower values of each predictor’s variable are represented by the red and blue colors in the figure. The probability of an ARI is higher at points that are in line with the red color and lower in blue color (Fig. 4).

**Fig. 5:**
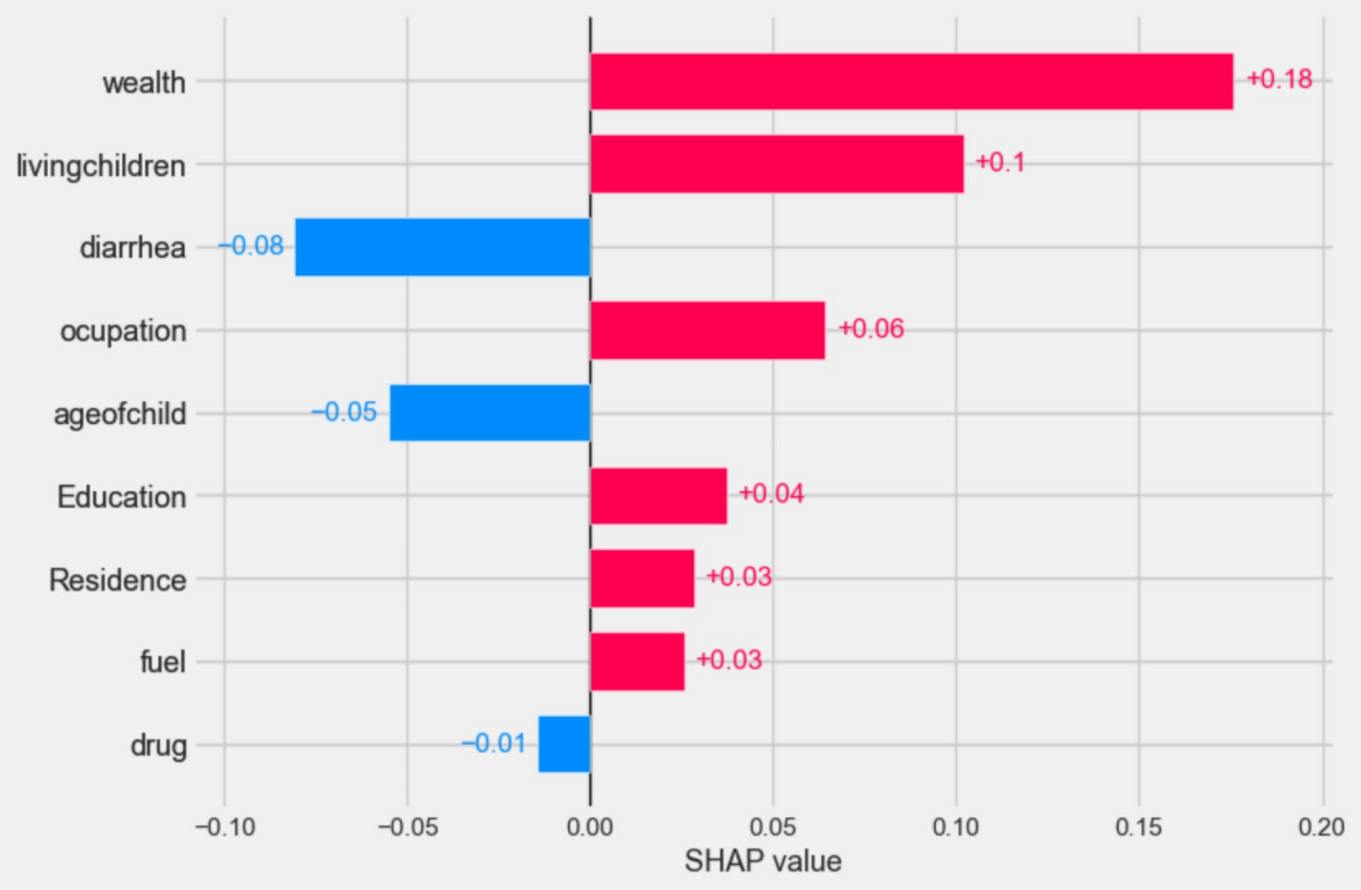
Bar plot result of global shaply value outcome generated by optimized random forest model.

In Fig. 5 bellow bar plot function were applied in order to identify the value and position of predictive feature for easiest Global predictors’ interpretation. According to the bar graph result of SHAP value of the model wealth index of family, number of living child with in the family, mothers of occupation, Mothers educational level, type of residence and fuel type for cooking are the strongest predictor respectively in positive whereas had diarrhea history, age of child and drug for intestinal parasite are negatively affected the predictor of ARIs (Fig. 5).

**Fig. 6:**
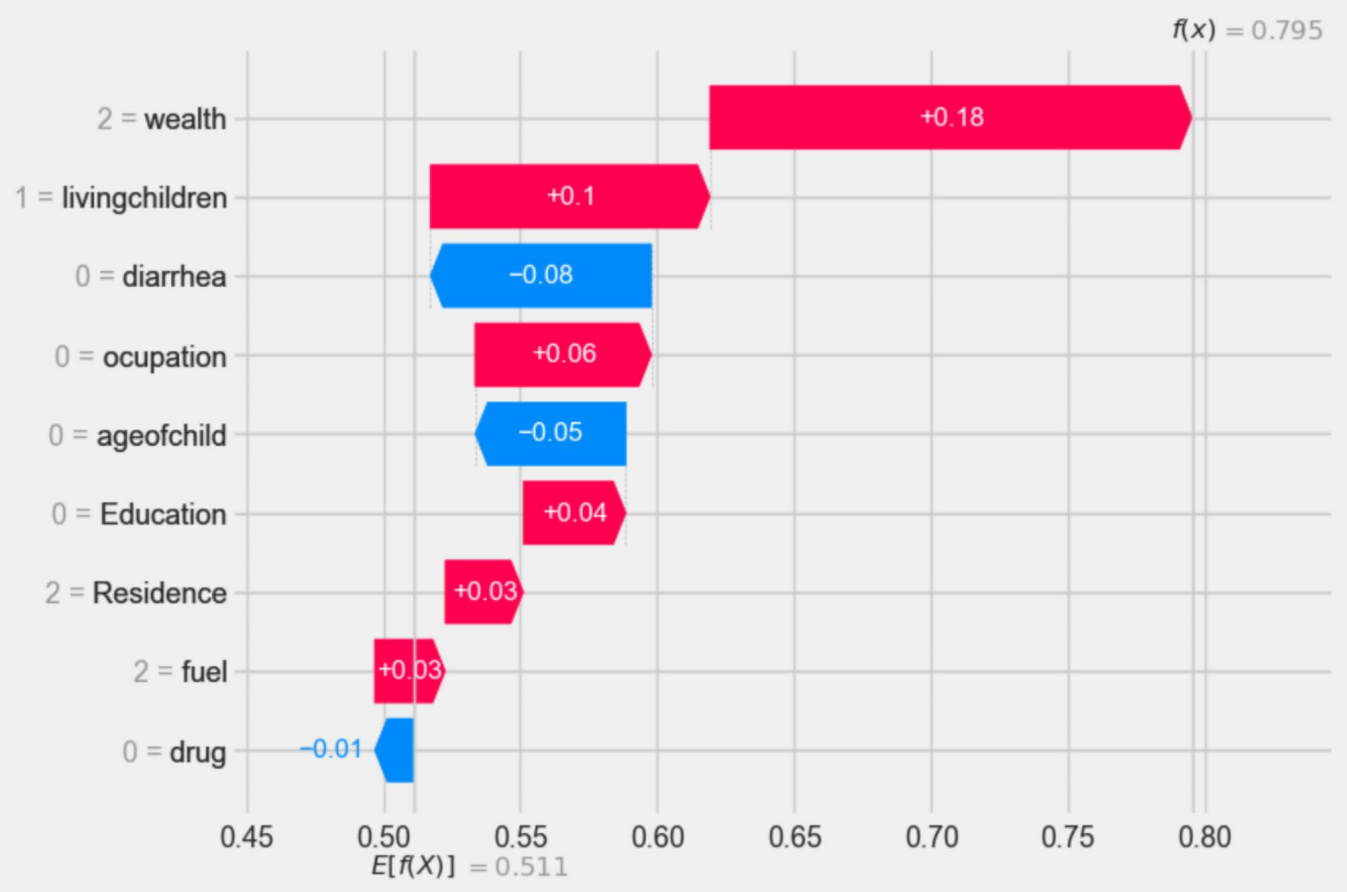
Waterfall plot displaying prediction of the ARI positive observation.

In Fig. 6 bellow, waterfall plots were utilized to provide an explanation for the model prediction pertaining to the ARI positive observations. The waterfall plots start with the expected value of the model output on the x-axis (E[f(X)] = 0.5), which is the initial prediction for the sample in question before taking feature contributions into account. Usually, this baseline prediction represents the dataset’s average or most frequent prediction. If the model output for a given observation is greater than this value (E[f(X)]), it indicates a positive class (i.e. ARI positive ), while results below this threshold indicate that there is “No ARI” in the negative class. As a result, for the first observation, the expected value output is moved to the final model output (f(x) = 0.795), which is categorized as positive class (had ARI), by the combination of the positive contributions (in red) and the negative (protective) contributions (in blue) and also it is used to identify local or individual predictability of the feature (Fig. 6).

According to waterfall result above families poorer wealth status (2 = wealth), number of children within the family 4–6 (1 = living children), being child without diarrhea history(0 = diarrhea), mothers who had working(1 = occupation), age of child less than 6 month(0 = age of child), no educated mother(0 = education), being live in rural(2 = residence), wood as a cooking material in the family(2 = fuel) and no intestinal parasites drug history (0 = drug), are high impact on the ARI prediction respectively either positively (represented by red color) or negatively(indicated by blue color).

## Discussion

This study provides a brief overview of the prediction of Acute Respiratory Infection (ARI) and its determining factors among children under the age of five in Amhara Regional state, Ethiopia, utilizing machine learning techniques. Seven machine learning algorithms were evaluated for their prediction accuracy and area under the curve (AUC). The algorithms tested include Random Forest, Decision Tree, Naive Bayes, K-Nearest Neighbors (KNN), Support Vector Machine (SVM), Logistic Regression, and Gradient Boosting. Among these algorithms, the Random Forest model demonstrated the highest prediction accuracies and AUC statistics, indicating its superior predictive ability compared to the other models employed in this study. Several factors were identified as important predictors of ARI among children under five, including wealth index, history of diarrhea, age of the child, mother’s education, number of living children in the household, mother’s occupation, intestinal drug usage, type of residence, and type of fuel used. In summary, this study highlights the effectiveness of machine learning algorithms in predicting Acute Respiratory Infection in young children. The Random Forest model, in particular, exhibited the strongest predictive performance, while identifying several key factors associated with ARI occurrence in this population.

The results obtained from the best-performing machine learning (ML) model align closely with those of the conventional logistic regression analysis. This similarity suggests that factors such as diarrheal history, wealth index, mother’s education, mother’s occupation, fuel type, and use of intestinal drugs significantly influence the occurrence of Acute Respiratory Infection (ARI) among children under five in Amhara region, Ethiopia. However, the ML models introduce some additional variables that were not identified by the traditional logistic regression analysis. These variables include the child’s age, the number of children still living at home, and the type of residence. This finding implies that ML models may uncover previously unknown insights or generate different variables that could be crucial for informed policy decision-making, which were not captured by conventional regression models. In summary, the ML models provide additional insights into the determinants of ARI among young children, highlighting the importance of variables such as age, household composition, and residence, in addition to the factors identified by logistic regression. This information can be valuable for policymakers in developing effective strategies to combat ARI in this population.

In this study, the study demonstrated that the Children’s Family Wealth Index is a predictor of ARI in children under the age of five. Being child’s family with poor wealth index has log odd of 0.18 or 1.20 odds of suffering from ARI (P-value: <0.01 95% CI: 1.17–1.22) as compared to their counterpart. The study conducted in Bangladesh lends weight to these conclusions^29^. Children under the age of five whose families were from lower socioeconomic classes also had a strong association with ARI^24,30–32^. This is because families with more money can usually afford to provide their kids with better nutrition and healthcare. Richer households can also reduce their kids’ exposure to dangers like tainted water and unhygienic surroundings. Other studies that indicated that the risk of ARI and diarrhea is increased at increasing poverty levels provide weight to these findings.

This study revealed that the number of living children in the same household is a significant predictor for Acute Respiratory Infection (ARI) among children under the age of five in Amhara Regional state, Ethiopia. The findings indicate that children living in households with a higher number of children by the log odds of 0.1 or 1.11 odds of suffering from ARI (P-value: <0.01 95% CI: 1.05–1.16) compared to those in households with fewer children. One possible explanation for this vulnerability is that having a larger number of children in the same household can make it challenging to manage and provide adequate health treatment. Additionally, access to a balanced diet or proper nutrition may be limited in such households, further contributing to the increased risk of ARI among children. In summary, the study highlights the relationship between the number of children in a household and the susceptibility to ARI in children under five. The challenges associated with managing a larger number of children in the household, as well as potential limitations in healthcare access and nutrition, may contribute to this increased vulnerability.

ARI (Acute Respiratory Infection) had a significant impact on young children under the age of five, particularly those who had a history of diarrhea. Interestingly, children who had not experienced diarrhea in the past were found to have a protective effect against ARI compared to children with a history of diarrhea, with a log odd of -0.08 or 0.92 odd of suffering from ARI (P-value: <0.01 95% CI: 0.88–0.98) as compared to the child with diarrhea history. A case-control study conducted in the North East Oromia zone of northeast Ethiopia and Zimbabwe^8,33^ revealed that children with a previous history of diarrhea were found to have a higher likelihood of experiencing Acute Respiratory Infection (ARI) compared to their counterparts, Similar investigations were conducted in Ghana^34^ and southwest Ethiopia^35^ confirmed this conclusion. A cross-sectional investigation conducted in Bangladesh^36^ revealed that children with a history of diarrhea were found to have a higher likelihood of developing Acute Respiratory Infection (ARI). The study suggested that this association could be attributed to decreased immunity in children with concurrent illnesses, such as diarrhea, making them more susceptible to ailments like ARI.

Additionally, this study demonstrated a substantial relationship between ARI and child age. In this study child whose age is less than six months are more protective than the older one by the log odd of -0.05 or 0.95 odd of suffering from ARI (P-value: <0.01 95% CI: 0.93–0.97) as compared with their counterparts. The findings align with previous research conducted in Indonesia, Uganda, and Bangladesh^37–39^, indicating that older children are more affected by Acute Respiratory Infection (ARI) compared to their younger counterparts. Additionally, studies conducted in India and the southeast of Brazil have shown that children in the younger age group are more likely to be hospitalized due to ARI^40,41^. One possible reason for older children being more affected by Acute Respiratory Infection (ARI) could be their close contact with diverse environments and individuals who may have coughs. This increased exposure to viruses and bacteria could potentially raise the likelihood of contracting ARI^42^. Another possible reason for younger children being more protected against ARI compared to their older counterparts could be attributed to the practice of exclusive breastfeeding. Breast milk has immune-boosting properties that can enhance the immunity of children^43,44^, making them less susceptible to ARI. Additionally, the adherence to vaccination practices for children under the age of five may also contribute to their enhanced protection against ARI.

According to this study, among children under the age of five, the mother’s profession was linked to ARI, children who had mothers without work are more affected by the log odd of 0.06 or 1.06 odd of suffering from ARI (P-value: <0.05 95% CI: 1.04–1.08) than children whose mother had occupation. According to EDHS 2011^45^, children in Pakistan^46^ who had working moms had a considerably greater chance of having ARI than children whose mothers did not work. Mothers play a significant part in childhood ARI, which might be the basis for the defense^47^. The third possibility is that working moms have been exposed to harmful chemicals, pollutants, or gases while at work, increasing the likelihood that they may infect their children^48^. Children become more susceptible to ARI when moms are working since they do not have enough time to nurse their kids^49^.

The mother’s educational level was found to be a predictor of acute respiratory infections (ARI) among children under five. Children with no educated mother were more affected by ARI, with log odds of 0.04 or 1.04 odd of suffering from ARI (P-value: <0.05 95% CI: 1.02–1.06) as compared to their counterparts. This finding aligns with previous research findings^50,51^. The association between a mother’s education and reduced risk of acute respiratory infections (ARI) in children under five can be attributed to several factors. Firstly, education equips mothers with the necessary knowledge and skills to navigate their surroundings, including healthcare facilities, and effectively collaborate with medical professionals. This enables them to better understand and follow treatment recommendations for their children. Secondly, educated mothers are more likely to prioritize hygiene practices and maintain a clean environment, minimizing the risk of exposure to pathogens that cause respiratory infections. They are aware of the importance of sanitation, proper hand washing, and a healthy living environment. Lastly, maternal education empowers women to have a greater influence over their children’s health choices. Educated mothers tend to make informed decisions regarding preventive measures, such as vaccinations, proper nutrition, and breastfeeding, which can enhance their child’s immune system and reduce the likelihood of ARI. Overall, the positive impact of maternal education on reducing ARI risk can be attributed to the tools and knowledge it provides to mothers, enabling them to effectively manage their child’s health, collaborate with healthcare professionals, adhere to treatment recommendations, and create a healthier environment for their children.

Even though, the importance of the variables is less compared with the other variables in this study, children living in rural residence by the log odd of 0.03 or 1.03 odd of suffering from ARI (P-value: <0.05 95% CI: 1.01–1.05) as compared to their counterparts. This result of the study is in line with the study done in Indonesia^37^, using wood as cooking fuel as cooking material in the household is affected by ARI with log odd of 0.03 or 1.03 odd of suffering from ARI (P-value: <0.05 95% CI: 1.01–1.05) as compared to their counterparts. This result of the study is in line with the study done in Ethiopia^52^ and having intestinal drug of child had protective from ARI with the log odd of -0.01 or 0.99 odd of suffering from ARI (P-value: ~0.05 95% CI: 0.97– 1.00) as compared to their counterparts. However the association of this result is not statically significant.

In comparison to classic regression models, the machine learning (ML) findings may appear less interpretable as they lack regression coefficients and the direction of impact. However, in practice, ML models can categorize or predict specific factors based on their significance in influencing acute respiratory infection (ARI) levels among children under the age of five in the current study. To determine the direction of these important factors, one can refer to existing empirical literature that employs traditional approaches. This allows for an understanding of the influence of these factors on ARI outcomes. Nevertheless, despite their potential lack of interpretability, machine learning methods are highly valuable in forecasting population health and other phenomena. They contribute to improved policy decisions by providing insights and predictions that can guide interventions and strategies to mitigate ARI in children and enhance overall health outcomes^53,54^.

## Conclusion

In this study, machine-learning algorithms were employed to develop a prediction model for acute respiratory infection (ARIs) in children under the age of five in Amhara regional state, Ethiopia. Through the use of design science methods, a suggested model was created using various homogeneous ensemble machine learning methods, including Random Forest, Decision Tree, Naive Bayes, KNN, SVM, Gradient Boosting, and Logistic Regression. Nine experiments were conducted, and the Random Forest algorithm demonstrated the highest performance, achieving 90.35% accuracy, 87.76% sensitivity, 92.93% specificity, 92.22% positive predictive value, and 88.83% negative predictive value.

By conducting ex-Additive SHAP value analysis on the best-performing algorithms, the researchers identified the key risk factors associated with ARI among children in the Amhara regional state, Ethiopia. The study revealed that the wealth index of the family, the number of children living in the same household, the mother’s occupation, the educational level of the mother, the type of residence, and the fuel type used for cooking were significantly correlated with ARI in this population. Furthermore, the research emphasized that children without diarrhea and a smaller number of children living in the same household are crucial factors for improving the health outcomes of children in the Amhara regional state, Ethiopia.

## Data Availability

The datasets used or analyzed during the current study are available from the corresponding author on reasonable request. The data was obtained with a link https://www.dhsprogram.com/data/dataset_admin/login_main.cfm.

## Abbreviations

ARI: Acute respiratory infection
AUC: Area under the curve
EDHS: Ethiopian Demographic and Health Survey
KNN: K-nearest neighbor
ML: Machine learning
NP: Negative predictive
PP: Positive predictive, RF, Random forest
ROCAUC: ROC curve
SHAP: Shapley Additive exPlanations
SPSS: Statistical Package for Social Sciences
SVM: Support vector machine
SMOTE: Systematic Minority Over Sampling Technique
